# Prioritising references for systematic reviews with RobotAnalyst: A user study

**DOI:** 10.1002/jrsm.1311

**Published:** 2018-07-30

**Authors:** Piotr Przybyła, Austin J. Brockmeier, Georgios Kontonatsios, Marie‐Annick Le Pogam, John McNaught, Erik von Elm, Kay Nolan, Sophia Ananiadou

**Affiliations:** ^1^ National Centre for Text Mining School of Computer Science, University of Manchester Manchester UK; ^2^ Cochrane Switzerland, Institute of Social and Preventive Medicine Lausanne University Hospital Lausanne Switzerland; ^3^ National Institute for Health and Care Excellence Manchester UK

## Abstract

Screening references is a time‐consuming step necessary for systematic reviews and guideline development. Previous studies have shown that human effort can be reduced by using machine learning software to prioritise large reference collections such that most of the relevant references are identified before screening is completed. We describe and evaluate RobotAnalyst, a Web‐based software system that combines text‐mining and machine learning algorithms for organising references by their content and actively prioritising them based on a relevancy classification model trained and updated throughout the process. We report an evaluation over 22 reference collections (most are related to public health topics) screened using RobotAnalyst with a total of 43 610 abstract‐level decisions. The number of references that needed to be screened to identify 95% of the abstract‐level inclusions for the evidence review was reduced on 19 of the 22 collections. Significant gains over random sampling were achieved for all reviews conducted with active prioritisation, as compared with only two of five when prioritisation was not used. RobotAnalyst's descriptive clustering and topic modelling functionalities were also evaluated by public health analysts. Descriptive clustering provided more coherent organisation than topic modelling, and the content of the clusters was apparent to the users across a varying number of clusters. This is the first large‐scale study using technology‐assisted screening to perform new reviews, and the positive results provide empirical evidence that RobotAnalyst can accelerate the identification of relevant studies. The results also highlight the issue of user complacency and the need for a stopping criterion to realise the work savings.

## 1 INTRODUCTION

Systematic reviews seek to answer specific research questions and form unbiased, evidence‐based conclusions by combining information from all relevant studies. They are used to compare treatments, diagnostic tests, health service organisations, prevention strategies, etc and to develop evidence‐based guidelines on health and social policy and clinical practice.^1^, ^2^, ^3^ Coarsely, a systematic review involves at least five stages: formulation of the research question and inclusion criteria, literature search to identify a set of possibly relevant references, initial relevancy screening based on reference and abstract, further screening based on the full text, and evidence extraction and synthesis of findings.

While all stages are resource‐intensive, designing literature database searches and performing abstract‐level screening are tasks that are increasingly time‐consuming due to the ever‐growing corpus of published literature.^4^ Information specialists must design search strategies that are sufficiently sensitive to gather all relevant studies while specific enough to limit the result size. This is especially challenging in public health, because the research questions are often broad and the inclusion criteria, expressed in terms of the traditional PICO framework (population, problem, patient; intervention; comparison, control, comparator; and outcome), involve broad definitions for populations and complex interventions. Furthermore, the definitions of interventions lack consistency across studies. As a result, literature searches for public health evidence often have low specificity and return large volumes of references to be screened at the title and abstract level.

Screening references is a lengthy process, and double screening with two reviewers is recommended to avoid missing references.^1^, ^5^ Given that the estimated screening time per reference (title and abstract) is between 30  seconds^6^ and 1  minute^7^ and the estimated time to discuss and resolve an inclusion disagreement between reviewers is approximately 5  minutes per reference, screening 5000 references will last between 83 to 125  hours per reviewer.^7^ The estimated costs are also considerable: amounting to £13  000 for a single review.^7^

This burden motivates the use of computational tools to assist manual screening to reduce the workload in cases of low specificity.^8^ For example, search tools can be used to select a small initial subset of the references sharing a certain characteristic, eg, the presence of a keyword or its synonyms. Furthermore, machine learning algorithms that learn from a human screener's decisions can be harnessed to prioritise the remaining unseen references by their predicted relevancy.^9^ With prioritisation, it may be possible to perform a partial screening and still identify the vast majority, for example, 95%, of the relevant references.^9^ Prioritisation may also enable different screening or review paradigms, such as living systematic reviews^10^, ^11^ or updates,^12^, ^13^  since more relevant references are found earlier than with manual screening.^14^

RobotAnalyst *
http://nactem.ac.uk/robotanalyst/.is a Web‐based screening system that leverages algorithms from information retrieval, text mining, natural language processing, and machine learning to assist reviewers in prioritising references and exploring a reference collection using automatic terminology extraction, topic modelling, and descriptive clustering. While RobotAnalyst has been purposefully designed to handle the challenges of terminological variation and low specificity in the large collections encountered with public health reviews, these challenges are ubiquitous and it can be applied to any screening task at the title and abstract level.

The potential benefit of incorporating machine learning into the systematic review toolkit has been showcased by numerous retrospective studies (simulations of the screening process using previously screened collections^9^, ^13^, ^15^, ^16^, ^17^, ^18^, ^19^, ^20^, ^21^, ^22^, ^23^, ^24^, ^25^, ^26^, ^27^, ^28^, ^29^, ^30^, ^31^) and some prospective studies^32^, ^33^ that have explored different machine learning approaches. Yet, before semi‐automated tools with functionality like RobotAnalyst become widely adopted within the systematic review community, there is a need for real‐world evaluation, user feedback, and discussion to understand the benefits and potential risks. While multiple software solutions^33^, ^34^, ^35^, ^36^ leverage machine learning prioritisation to assist review screening, published evaluations have mainly been limited to cross‐validation of previously completed reviews. There is a lack of studies where the screening—from start to finish—is completed with computer‐assisted prioritisation. Real‐world evaluations of semi‐automated tools' performance by reviewers are necessary to confirm their theoretical benefits and ensure that they actually support review and guideline development.^37^  In particular, real‐world evaluation can be used to track metrics such as decision accuracy and time per decision across the screening process. This is important to assess additional aspects, such as software interface design and real‐time operation.

We have conducted an evaluation of RobotAnalyst for technology‐assisted screening at two sites. Multiple reviews were performed for public health guidelines and new surveillance reviews within the National Institute for Health and Care Excellence (NICE). †
NICE conducts systematic reviews to identify effective interventions and inform the development of public health guidelines http://www.nice.org.uk/guidance.Another review was conducted by reviewers from the Cochrane Switzerland group, ‡
http://swiss.cochrane.org/.at the Institute of Social and Preventive Medicine (IUMSP), Lausanne University Hospital, to inform patient safety and quality of hospital care. Results from both sites highlight the ability of RobotAnalyst to prioritise relevant references early in the screening process.

In addition to prioritisation, RobotAnalyst offers functionality for the exploration of reference collections via descriptive clustering^38^, ^39^, ^40^, ^41^ and topic modelling.^42^, ^43^, ^44^, ^45^ These techniques take text documents, such as reference abstracts, and divide them into a set of clusters or topics, each associated with a subset of references that share similar vocabulary. References with the same topic or cluster may be thematically related even if there are no common keywords they all share. Furthermore, the topic proportions of each reference can be used to find related references, and as a feature representation for machine learning.^26^, ^29^, ^36^ Primarily, clusters and topics, supplemented with automatically generated descriptions,^40^, ^41^, ^46^ allow reviewers to explore the thematic coverage^44^, ^47^ and locate relevant references,^48^, ^49^ without having to explicitly form keyword queries, within diverse collections. Searching and screening diverse collections are especially useful for supporting development of public health guidelines that involve multiple complex questions. Analysts from NICE have performed an evaluation of the coherence of RobotAnalyst's clustering and topic modelling and the descriptiveness of the keyword lists.

In summary, the key contribution of RobotAnalyst is the combination of active learning prioritisation with content, metadata, and topic and cluster‐based search. Reviewers can use the search capabilities to identify the initial set of inclusions and exclusions, before using active learning to prioritise. This is ignored in other evaluations based on cross‐validation of previously completed reviews. Furthermore, during screening, the inclusion ranking itself can be filtered using Boolean queries based on specific terms, clusters, or topics. This human‐in‐the‐loop ranking thus augments purely machine prioritisation.

## 2 RELATED WORK

The earliest study of machine learning to emulate the inclusion decisions for systematic reviews was the work of Cohen et al.^9^ Previously, machine learning had been demonstrated to be as effective for retrieving general categories (therapy, diagnosis, aetiology, and prognosis) of high‐quality studies for evidence‐based medicine literature^50^, ^51^ as hand‐tuned Boolean queries.^52^ Subsequent studies^15^, ^16^, ^17^, ^18^, ^19^, ^20^, ^21^, ^22^, ^23^, ^24^, ^25^, ^26^, ^27^, ^28^, ^29^, ^32^, ^33^, ^36^ have explored different feature spaces (words or multiword patterns, MeSH terms from PubMed metadata, unified medical language system (UMLS) for nomenclature, and topical or thematic features) and machine learning models and techniques such as naive Bayes, support vector machine (SVM), and logistic regression. Others have incorporated out‐of‐topic inclusions^53^ and unscreened references.^30^, ^31^

Most previous studies of machine learning for systematic reviews have a fixed training and test set of references. In these cases, a user screens a portion of references (either random or based on publication year for an update); the machine learns from the screened portion and predicts the relevant references within the remainder of references (the test set). Finally, only the references predicted as relevant are screened manually by a human reviewer. In this scenario, two reviewers (human and machine) have screened every inclusion. A similar scenario involves two human reviewers that each screen half of the collection to train two independent classification models. Each model provides relevancy predictions on the other half, and discrepancies are resolved by the humans.^18^

A key issue with these scenarios is the low specificity within the training set. This poses a problem since identifying all or nearly all of the inclusions is essential for systematic reviews, but off‐the‐shelf machine learning algorithms offer predictions under the assumption that misclassifications, eg, predicting an inclusion instead of an exclusion or vice versa, are equally unwelcome. This is not the case in systematic reviews, where unnecessary inclusions during abstract‐level screening (by being overly inclusive) can later be discarded, while missing relevant references violates the purpose of systematic reviews.^1^ Without adjustment, the classification model may perform poorly with imbalanced samples. To overcome this, principled adjustments and various ad hoc techniques, such as subsampling or reweighting, have been explored.

Active learning^54^, ^55^, ^56^ is the process of using a classification model's predictions to iteratively select training data. It provides an alternative scenario for prioritising the screening process from the beginning to the end, which naturally ameliorates the imbalanced sample problem. After training with a small set of references screened by a human, active learning proceeds by prioritising references based on their predicted relevancy and the confidence of this prediction. One objective of active learning is to select training examples that improve the model as quickly as possible such that it can eventually be applied to the remaining references. In this case, the references which have the lowest confidence in their model predictions are screened first.^22^, ^23^, ^26^, ^30^, ^31^, ^54^ Another approach is relevancy‐based prioritisation, where the references with the highest probability of being relevant are screened first^24^, ^26^, ^29^, ^31^, ^33^ (a process known as relevancy feedback^57^ or certainty‐based screening^26^). Essentially, active learning uses new screening decisions made by the user to improve the prioritisation throughout the process. Furthermore, active learning naturally handles the imbalanced sample problem by including references with a substantial chance of being relevant.

In the rest of this section, we review screening systems currently used in applications for systematic reviewing. Prioritisation performance for some of these systems has been measured, but the nature of the evaluation settings varies.

EPPI‐reviewer^34^ is a tool for reference screening available through a Web‐based interface for a subscription fee. §
https://eppi.ioe.ac.uk/cms/er4/.It contains automatic term recognition using several methods, including Termine from the National Centre for Text Mining,^58^ which, as described in the EPPI‐reviewer user manual, ¶
https://eppi.ioe.ac.uk/CMS/Portals/35/Manuals/ER4.7.0%20user%20manual.pdf. could be used to find relevant references based on terms found in previous inclusions. References can also be clustered using Lingo3G software. #
https://carrotsearch.com/lingo3g/.Reference prioritisation is not generally available to all users, but it has already been tested for scoping reviews,^59^ which differ from systematic reviews by taking into accounts much larger sets of possibly eligible references and having eligibility criteria developed iteratively during the process. EPPI‐reviewer was used in two scoping reviews, containing over 800  000 and 1 million references, and provided substantial workload reduction (around 90%). One should note though that because of collection sizes, not all references were manually screened, so recall was estimated using random samples from the whole reference set.

Specifically designed for facilitating screening based on active learning, Abstrackr^33^ is a free online open‐source tool ‖
http://abstrackr.cebm.brown.edu/.that uses the dual supervision paradigm, where the classification rules are not only automatically learned from screening decisions but also provided explicitly by users as lists of words, whose occurrence in text is indicative for reference inclusion. Another interesting extension is collaborative screening, which takes into account different levels of experience and costs of reviewers working on the same study in an active learning scenario.^60^, ^61^ The underlying classifier is an SVM over n‐grams (word sequences). A prospective evaluation using relevancy‐based prioritisation was performed by an assistant, who used decisions by a domain expert to resolve dubious cases. An independent evaluation^62^ was performed on four previous reviews (containing 517, 1042, 1415, and 1735 references). In this case, only the inclusions were evaluated by a reviewer, while exclusions were judged by verifying whether they were present in the published reviews (ie, the references were included after full‐text screening). The reported work saved was 40%, 9%, 56%, and 57%, respectively.

SWIFT‐Active Screener **
https://www.sciome.com/swift-activescreener/.is a Web‐based interface for systematic reviews with active learning prioritisation.^36^ Similar to RobotAnalyst, it uses bag‐of‐words features (the counts of distinct words within the title and abstract) and the topic distributions estimated by latent Dirichl et allocation,^43^, ^63^ and prioritises references using a logistic regression model. The differences are SWIFT's inclusion of MeSH terms and RobotAnalyst's use of a linear SVM for the classification model rather than logistic regression. SWIFT‐Active Screener implements a separate model to predict the number of inclusions remaining to be screened, which can be used as a signal for the reviewer to stop screening. The system is interoperable with a related desktop application, SWIFT‐Review, ††
https://www.sciome.com/swift-review/.which is freely available. A cross‐validation evaluation^36^ across 20 previously completed reviews, including 15 from Cohen et al,^9^ has shown consistent work saved over sampling.

Rayyan^35^ is a free Web application ‡‡
https://rayyan.qcri.org/.for systematic review screening. The machine learning model^28^ is an SVM‐trained classifier that uses unigrams, bigrams, and MeSH terms and suggests relevancy using a 5‐star system. It was evaluated on data from Cohen et al,^9^ and a pilot user study on two previously completed Cochrane reviews (273 and 1030 references) was undertaken for qualitative evaluation. The interface provides a simple tool for noting exclusions reasons and supports visualisation of a similarity graph of references.

Another Web‐based system for screening references is Colandr. §§
http://www.colandrcommunity.com/.The system has an open‐source code base and uses a linear model that is applied to vector representations of references based on word vectors.^64^, ^65^

Besides screening prioritisation, there are other text‐mining tools to assist study selection for systematic reviews.^66^ For some systematic reviews, the inclusion criteria dictate that the reference describes a randomised control study or that the study uses certain methodologies (eg, double‐blinding) to ensure quality. Tools to automatically recognise these^69^,^67^, ^68^, ^69^, ^70^, ^71^ can be used to generate tags to filter references. Study selection can also benefit from fine‐grained information extraction from full article text, eg, to find sentences corresponding to PICO criteria elements,^72^ or from efforts to automatically summarise included studies.^66^

In summary, numerous studies have evaluated automatic classification for systematic reviews; some of which have been implemented within end‐user systems, but their evaluations have been limited to either simulations involving previously completed reviews or partial reviews that have not been verified by complete manual screening. To the best of our knowledge, our work is the first large‐scale user‐based evaluation that performs new screening tasks from start to finish.

## 3 METHODS

In this section, we firstly describe RobotAnalyst's core functionality and implementation. An overview of the user interfaces is presented in Appendix A. Secondly, we describe the evaluation methodology.

### 3.1 System functionality

**Figure 1:**
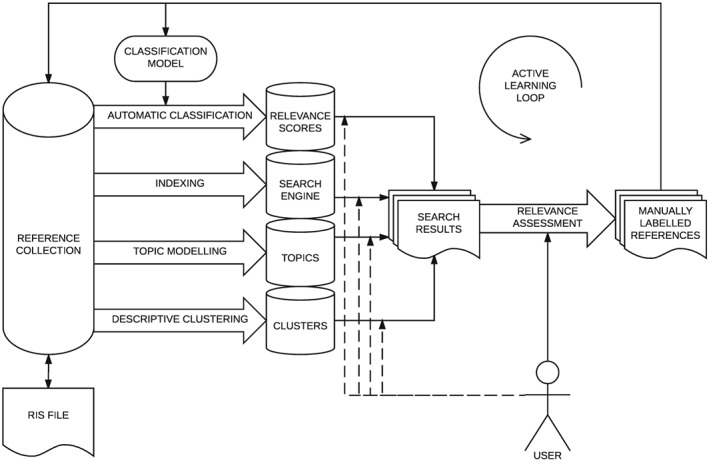
An information flow diagram of the information processing and user interaction available in RobotAnalyst

To support screening, RobotAnalyst's interface allows a user to combine searches based on content, clusters, or topics with active learning prioritisation. As shown in Figure 1, the input to the screening process is a collection of references, and the output is the subset of relevant references included for further review or synthesis. The user's screening decisions inform the classification model, which in turn affects the prioritisation of references in the active learning loop.

An initial batch of references (selected at random or via a focused search) with manual screening decisions is used to train an initial classification model, which recommends further references for screening. If a user chooses to screen them, the decisions, in turn, are used to train a better classification model, which will suggest further references. In every iteration of this active learning loop, a classifier is improved by having more training data and, as a result, can provide better predictions and suggestions.

Although a reviewer can use the system's relevance scores in many ways, relevancy‐based prioritisation,^26^ where the references the system deems as the most likely to be relevant are screened, is the suggested approach. Prioritising the relevant references earlier in the screening process^31^ is considered beneficial for gaining an understanding of the area^14^ or in cases when the reviewer does not plan to screen the whole collection.^9^

As soon as new screening decisions have been made, the user can trigger the retraining process to update the system's predictions and the inclusion confidence for each reference by using the new decisions to train the classification model. Updating the model frequently may provide more accurate predictions for prioritising the relevant references. However, to avoid excessive computation and to provide a stable user experience, the training is manually triggered by the user, with the system issuing a reminder after 25 decisions without an update.

An important feature of RobotAnalyst is its flexibility. A user can choose to select references for screening using the search functionality, the system's inclusion confidence for prioritisation, or a combination of both. Since model retraining is triggered manually, users can focus on assessing a large portion of the collection before rebuilding the model or choose to make frequent updates to get the most accurate predictions. The system does not attempt to substitute for the reviewer but rather to aid the reviewer throughout the screening process.

### 3.2 System implementation

RobotAnalyst is implemented as a server‐side Web application, which means that all screening process data are stored and processed on a remote server while the interface is accessible as an interactive Web page using a standard Web browser.

When a user uploads a reference collection (in RIS file format,^73^ which describes each reference by its title, abstract, and metadata), each reference is converted into two text documents corresponding to title and abstract. These documents are subsequently processed by

1. text‐mining and automatic term detection,
2. generating a topic model based on contents of abstracts to find references that discuss similar topics while allowing variations in vocabulary,
3. creating a feature representation for descriptive clustering to divide the collection into groups of similar references that can be browsed via lists of associated keywords,
4. and building the feature representations for the active‐learning classification model used for predicting the relevant references and prioritising them.

The original content and metadata along with the extracted information (topics, clusters, and terms) are indexed for collection‐specific search using Apache Solr search server. ¶¶
http://lucene.apache.org/solr/.The Solr database also stores the user's screening decisions and user‐supplied notes along with the machine‐generated classification predictions.

The text‐mining pipeline begins by passing the title and abstract through the GENIA tagger,^74^ which records the part of speech of each word and its lemma (the word's base form before affixing plurals, tense markers, etc.). To identify multiword terms for searching, we use the C‐value method implemented in Termine to identify candidate terms in each reference based on multiword noun phrases.

For topic modelling, we use the latent Dirichlet  allocation (LDA) model,^43^ a standard model that assumes each text is a mixture of topics with the proportion of topics varying between the texts. We use the MALLET^63^ toolkit to create an LDA model with 300 topics ##
The topic proportions are used as features that smoothly consolidate the occurrences of words from the same topic. In practice, the optimal number of topics depends on the collection and screening task. If too few topics are used, the topics would not be sufficiently specific to improve the prioritisation. If too many are used, they would not provide any advantage over the word occurrences themselves. To arrive at 300 topics, we had followed previous work^26^, ^29^ and the MALLET user guide, which suggests between 200 to 400 topics.based on the text from the titles and abstracts (prior parameters are set as $\alpha =\frac{1}{300}$ and β  =  0.01 and optimised every 50 iterations). The LDA model has multiple uses: It is used as a visual‐aided search interface for selecting references according to topics, for a similarity measure to compare references via the cosine distance between their topic vectors, and as additional input features for the classifier. For the former, each topic is described by the set of the 5 most frequent words and the set of 45 references most associated to it.

For descriptive clustering, we use spectral clustering of the documents (title and abstract combined) to form the clusters and a statistical selection process to determine a set of words and multiword terms that succinctly describe each cluster.^41^ Spectral clustering^75^ operates on the cosine similarities between the bag‐of‐words vector representation of the abstracts and titles using the term frequency inverse document frequency (TF–IDF) weighting.^76^ The vocabulary is limited to words that occur at least five times in the collection and are not present in the stop‐words list. The spectral clustering algorithm relies on the truncated eigen‐decomposition of an N‐by‐N matrix, where N is the number of references in the collection. To scale to large collections, this similarity matrix is never explicitly created; instead, the matrix‐vector multiplications required for the eigen‐decomposition are computed as a series of sparse matrix multiplications. The spectral representation is clustered using spherical k‐means, 10 replicates, a maximum of 100 iterations, and the scalable k‐means++ oversampling initialisation algorithm^77^ with the parameters l  =  2k and r  =  5, where k is the number of clusters.

The list of keywords (both words, lemmatised words, and terms identified using Termine) used to describe each cluster are selected as the most informative features for the cluster. Specifically, the algorithm firstly selects keywords positively correlated with the cluster and then uses the conditional mutual information maximisation criterion^78^ for greedy forward selection with redundancy reduction. The number of keywords used for each cluster is selected by statistical model order selection using the Bayesian information criterion^79^ after fitting a model to predict the cluster membership based on the presence of the keywords.^41^ The keywords for each cluster are sorted based on their coefficient weights. A user can select the number of clusters from the multiples of five up to 100 clusters.

The system's inclusion confidence is provided by a binary classification model that can be updated after each screening decision using all prior decisions as training data. As input to the classification model, each reference is represented as a vector of features corresponding to the count of words occurring in the title and abstract and also the topic model proportions. Using the inferred topics as features has been previously shown to improve accuracy in screening prioritisation.^26^ Specifically, references are represented by three sets of features:

1. an L2‐normalised ‖‖
This ensures the sum of the squared values of the feature vector equals 1.bag‐of‐words representation of the title based on TF‐IDF scores of all lemmatised words not present in the stop‐words list,
2. an analogous bag‐of‐words representation for the abstract, 
3. and the topic‐proportion vector estimated by Gibbs sampling from the LDA model.

Past screening decisions (references labelled for either inclusion and exclusion) provide the training examples for a linear model fit with an L2‐regularised L2‐loss function using the dual formulation of the support vector classifier implemented in LIBLINEAR^80^ with the default parameter values: constraint violation cost parameter C  =  1 and stopping criterion ε  =  0.1. By design, a support vector classifier can handle cases when the number of features is greater than the number of references, which is typically the case with a bag‐of‐words representation. The classification model is applied to the entire set of references and the output values are converted to inclusion confidence values (between 0 and 1) by applying the logistic function. Finally, each confidence value is converted to either an inclusion or exclusion prediction by applying a user‐chosen threshold. The time necessary to update a model depends on the total number of labelled references. It is shorter (a few seconds) at the beginning of screening and longer towards the end of a screening process. For example, training on 5000 references can be completed in 1  minute. Furthermore, a user can continue to screen references using the current prioritisation after triggering the model update process, avoiding any lost time.

### 3.3 Evaluation

To assess the usefulness of RobotAnalyst for accelerating screening, we performed a real‐world evaluation by asking reviewers at two institutions to use the system to perform screening tasks. During the evaluation period (reviews were started on or after September 2015 and completed by August 2017), 22 collections were screened completely, ie, a reviewer made a relevance decision for every reference. All collections were used with no post hoc selection. The collections screened vary in many aspects as detailed in Table 1. Most of the screening tasks were performed at NICE; the remaining and two were conducted at IUMSP to inform patient safety and quality of hospital care for older patients in Switzerland. The collections differed greatly in size, ranging from a small collection of 86 references to a large one of almost 5000. The percentage of relevant references varied from 0.28% (just 6 out of 2148) to almost 30%. The extremely low relevancy rate represents a challenge for machine learning classification, since the prioritisation can only be used once at least one relevant reference has been screened.

**Table 1:** Details of reference collections used in the evaluation experiments, including their topical areas (surv denotes surveillance reviews), origin (with relevant NICE guideline if applicable), overall size, and percentage of relevant references (averaged in case of parallel reviews)

**Collection**	**Topic**	**Origin**	**Size**	**Specificity, % **
TUB	Tuberculosis	NICE ^a^	4678	2.42
BC	Behaviour change: individual approaches	NICE ^b^	1502	13.72
BC‐S	Behaviour change: individual approaches (surv)	NICE ^b^	937	21.66
BC‐C	Choice architecture in behaviour change (surv)	NICE ^b^	959	15.33
WC‐D	Walking and cycling (surv, database search)	NICE ^c^	304	27.30
WC‐C	Walking and cycling (surv, citation search)	NICE ^c^	468	12.18
WC‐F	Walking and cycling (surv, focused search)	NICE ^c^	86	9.30
PAP	Physical activity and pregnancy	NICE	320	11.88
WGP	Weight gain and pregnancy	NICE	110	11.82
PW‐S	Preventing excess weight gain (surv, self‐weighing)	NICE ^d^	157	8.28
PW‐E	Preventing excess weight gain (surv, eating patterns)	NICE ^d^	719	5.15
WM	Weight management (surv)	NICE ^e^	665	29.62
SH	Sexual health	NICE ^f^	3760	1.36
QSH	Quality and safety in hospitals	IUMSP	4964	18.63
LD	Learning difficulties	NICE	2148	0.28
OCM	Osteoarthritis: care and management (surv)	NICE ^g^	2986	15.00
HB	Hepatitis B: diagnosis and management (surv)	NICE ^h^	1523	3.81

^a^Guideline: https://www.nice.org.uk/guidance/ng33.

^b^Guideline: https://www.nice.org.uk/guidance/ph49.

^c^Guideline: https://www.nice.org.uk/guidance/ph41.

^d^Guideline: https://www.nice.org.uk/guidance/ng7.

^e^Guideline: https://www.nice.org.uk/guidance/ph47.

^f^Guideline: https://www.nice.org.uk/guidance/ng68.

^g^Guideline: https://www.nice.org.uk/guidance/cg177.

^h^Guideline: https://www.nice.org.uk/guidance/cg165.

#### 3.3.1 Screening tasks

Two experiments were conducted: controlled and unconstrained. In the controlled experiment, two collections, Tuberculosis and Behaviour change, were each screened by three independent reviewers, following procedures using a defined subset of the system's functionality:

1. screen all references using relevancy‐based active learning (AL);
2. choose topics whose descriptive keywords match a user's keyword list defined a priori, screen all of these topics' references, and then continue with the rest of the collection in random order (topics);
3. or a combination of the above procedures: firstly, screen references from relevant topics, then continue according to relevancy‐based active learning (topics + AL).

The aim of these experiments was to compare the performance of these techniques in prioritising relevant references.

In the unconstrained experiments, reviewers, having been familiarised with the system's capabilities, used the system to perform various new abstract screening tasks. The reviewers were free to use any features of the system they found helpful in finding relevant references (descriptive clustering was not enabled).

A total of 16 screenings were performed in this manner without controlling how the reviewer performed the screening. By examining the database records, we could determine which screenings were AL‐based, ie, when reference selection was driven primarily by relevancy‐based prioritisation with other search functions used either very infrequently. Even if the screening was AL‐based, at the beginning of the task, the reviewer must use other capabilities or use the default sorting order, which was alphabetical by author name. With the unconstrained experiments, the baseline is screening from a random order.

One of the collections (Quality and safety in hospitals) was screened four times. Two reviewers screened without any prioritisation using Covidence ‡‡‡
https://www.covidence.org/.as the systematic review software. Then two other reviewers (one junior and one senior) used RobotAnalyst. The conflict‐resolved decisions from the first two reviewers serve as a baseline decision set, allowing us to assess the performance of the reviewers using prioritisation.

#### 3.3.2 Performance measures

The main objective is to prioritise the references such that the relevant references are identified first. To quantify the performance of the prioritisation, we use two measures: work saved over sampling at 95% (WSS@95%) and the area under the recall curve (AUR). Both measures are based on recall, which is the proportion of all relevant references identified. Recall can only be computed once the collection has been fully screened.

Specifically, WSS@95% measures the percentage of the collection that does not need to be screened if the reviewer were to stop screening upon achieving 95% recall, †††
In practice, it is not possible for a reviewer to know exactly when the desired recall has been achieved.compared with screening in random order.^9^ Precisely, 

(1) $$\begin{array}{ccc} \text{WSS}\text{@95\%} & =0.95-\frac{\text{TP}(i_{\text{R95}})+\text{FP}(i_{\text{R95}})}{N} & \\ & =0.95-\frac{i_{\text{R95}}}{N}, \end{array}$$

where N denotes the number of references, while TP(i) and FP(i) are the number of relevant and irrelevant references, respectively, found after screening i references (TP(i) + FP(i)  =  i), and i_R95_ denotes the number of references screened when 95% recall is firstly achieved.

(2) $$i_{\text{R95}}=\min_{\begin{array}{c} i\in \{1,\text{⋯},N\}\\ \text{recall}(i)\geq 0.95 \end{array}}i$$

(3) $$\text{recall}(i)=\frac{\text{TP}(i)}{\text{TP}(i)+\text{FN}(i)},$$

where FN(i) is the number of relevant references that have not been screened. During the initial screening, this number is unknown; consequently, WSS@95% can only be computed after the entire collection is screened.

To test the significance of the prioritisation, we use an exact test based on the distribution of WSS@95% under the assumption of a random ordering of the references. The probability that 95% recall is achieved after screening i references (ie, TP(i)  =  r  =  ⌈0.95R⌉ inclusions, where R is the total number of relevant references) is given as

(4) P(iR95=i)=N−Ri−rRr−1Ni−1R−r+1N−i+1.

The first term in the expression corresponds to the probability, given by the hypergeometric distribution, of a sample of i − 1 references (taken without replacement) having r − 1 inclusions, and the second term is the probability of observing the rth inclusion as the ith reference. The Pvalue is the probability of achieving 95% recall with i_R95_ or fewer references under the null hypothesis.

WSS@95% focuses on the workload at the fixed moment when 95% of relevant references are found, but depending on the review type, eg, scoping reviewing and update, the workload required at different recall levels may be more important. For example, a reviewer may be interested in how quickly the assisted screening can find 10% or 99% of the relevant references. This motivates the AUR metric, which averages the workload across all recall levels^81^. AUR is calculated as

(5) $$\text{AUR}=\frac{1}{N-\frac{1}{2}R}\sum_{i=1}^{N}\frac{\text{TP}(i)}{\text{TP}(i)+\text{FN}(i)}.$$

Because of the normalisation factor $N-\frac{1}{2}R$, the optimal value of AUR equals 1 for perfect prioritisation, ie, when all relevant references are screened before any exclusions.

## 4 RESULTS

**Figure 2:**
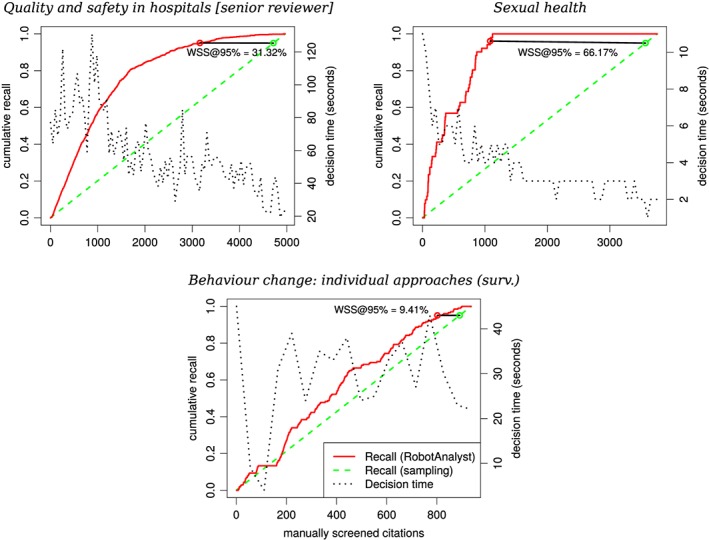
Cumulative recall curves and median decision times for three screening tasks. The times are smoothed by using the medians within a sliding window of 51 interdecision intervals. A graphical depiction of WSS@95% is shown as the difference between the recall curve and recall expected under a randomly sampled ordering of the references

Tables 2 and 3 show the evaluation results of the controlled and unconstrained experiments, respectively. In the cases when reviewers used the system's suggestions (AL‐based), the WSS@95% is significantly better than random ordering (exact test, significance level of 0.01). WSS@95% varies greatly between collections (from 6.89% to 70.74%), but the average gain is substantial (42.97%). We can see that the largest gains are achieved for tasks with very low specificity, ie, 1% to 3%, while tasks with more relevant references, ie, 30%, have lower values for work saved. For example, Figure 2 shows the recall and decision time across the whole process for a systematic review on Quality and safety in hospitals  (senior reviewer ) at IUMSP and a NICE guideline on Sexual health.

**Table 2:** Results of the controlled experiments performed on two reference collections, each screened using three procedures in parallel, with performance measured using WSS@95% and AUR metrics^a^

**Collection**	**WSS@95%**	**AUR**	**Strategy**
TUB	* 70.74%	0.9078	AL only
	* 69.67%	0.9196	topics + AL
	* 11.65%	0.7699	topics only
BC	* 29.89%	0.7983	AL only
	* 46.53%	0.8040	topics + AL
	‐1.80%	0.4729	topics only

^a^Values of WSS@95%, which were significantly greater than expected by random sampling (exact test, significance level of 0.01), are starred.

**Table 3:** Results of the unconstrained experiments performed, each involving screening a collection by a junior or senior reviewer using all the features of the system, with performance measured using WSS@95% and AUR metrics, grouped by whether relevancy‐based screening (AL‐based) prioritisation was used throughout ^a^

**Collection**	**Reviewer**	**WSS@95%**	**AUR**	**AL‐based**
BC‐C	Senior	* 6.89%	0.7276	Yes
WC‐D	Senior	* 29.54%	0.8477	Yes
WC‐C	Senior	* 22.35%	0.7904	Yes
PAP	Senior	* 40.63%	0.8398	Yes
WGP	Senior	* 36.82%	0.7893	Yes
PW‐S	Senior	* 63.15%	0.8285	Yes
PW‐E	Senior	* 38.81%	0.8369	Yes
WM	Senior	* 23.72%	0.8374	Yes
SH	Senior	* 66.17%	0.8858	Yes
QSH	Junior	* 39.84%	0.8914	Yes
QSH	Senior	* 31.32%	0.8818	Yes
LD	Senior	* 50.45%	0.9058	Yes
OCM	Senior	* 63.99%	0.9377	Yes
BC‐S	Senior	* 9.41%	0.6519	No
WC‐F	Senior	8.95%	0.5244	No
HB	Junior	‐3.62%	0.7347	No

^a^Values of WSS@95% which were significantly greater than expected by random sampling (exact test, significance level of 0.01) are starred.

In both cases, recall increases more rapidly than would be expected with random sampling and the decision time decreases when the number of relevant references diminishes in later stages of the process. Gains in both measures are more prominent when the relevant references are rarer such as *Sexual health*, with 1.36% specificity, as compared with *Quality and safety in hospitals* with 18.63% specificity. In fact, in the former case, 100% recall is achieved after screening just 29.84% of the collection. In contrast, when reviewers did not use AL‐based prioritisation, eg, *Behaviour change: individual approaches (surv)* as also shown in Figure 2, the gains measured by *WSS*@95% are lower or nonexistent.

**Figure 3:**
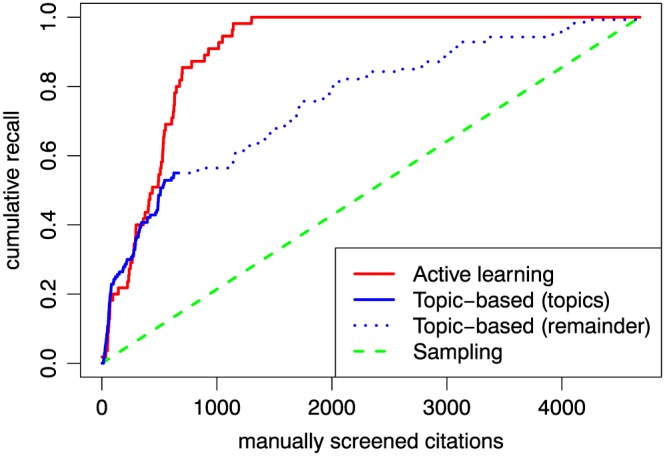
Cumulative recall curves for the Tuberculosis collection when using active learning versus topic‐based screening at the beginning followed by random sampling for the remainder

When reviewers relied on manual keyword or topic‐based search (not AL‐based), *WSS*@95% does not show gains but the *AUR* values are positive (values above 0.5). This is because these techniques enabled a user to find more relevant references within the search results that matched a topic or keyword than with random sampling at the beginning, which increases the AUR. Subsequently, after these returned results were screened, reviewers defaulted to random sampling and the early positive effect is less apparent with *WSS@95%*. We can rely on controlled experiments to verify this: for example, Figure 3 compares recall curves for two screening processes of the same collection (*Tuberculosis*): one was based on active learning, and the other one relied on browsing discovered topics. At the beginning, while the reviewer was able to choose references belonging to topics that seemed relevant (solid line), the increase in recall is similar to the one resulting from active learning‐based screening. However, when those topics were depleted (there are only 45 references per topic, and some may belong to multiple topics), the remainder of the collection was screened with no prioritisation (dotted line), which resulted in a slower return of relevant references. On the same collection, the third reviewer started by screening topics and then proceeded to active learning and achieved essentially the same performance, while this combination outperformed strictly active learning for the *Behaviour change*.

The controlled experiment results also show that topic modelling can be effectively used in the initial stages of screening (topics + AL) without degrading the performance versus strictly AL. Using topic‐based screening to kick‐start the classifier had either a neutral (*Tuberculosis*) or positive (*Behaviour change*) effect.

Although not yet evaluated for its prioritisation performance, descriptive clustering provides a topical organisation of all references within the collection enabling a reviewer to screen initially by clusters instead of topics. Furthermore, once a model has been trained, it can be used to prioritise the clusters by relevance and also prioritise the references within a cluster. An evaluation of the quality of descriptive clustering is given in Appendix B.

**Figure 4:**
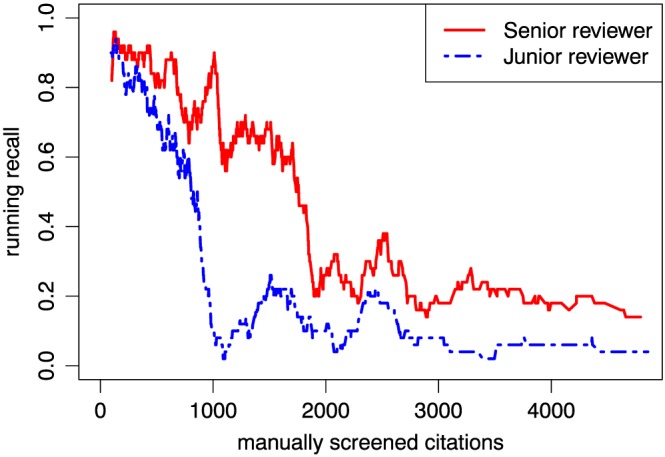
Running recall curves for the Hospital care quality collection, computed by comparing the decisions made by the senior and junior reviewer at a given stage of the process to a baseline decision set (see explanation in text)

Finally, we examine the performance of screening with prioritisation versus baseline screening. Figure 4 shows the screening performance across the *Quality and safety in hospitals* collection by a senior and junior reviewer in terms of running recall, which is the recall with respect to the baseline decision set measured in a moving window of 50 relevant references. For both reviewers, the recall is lower (more relevant references in the baseline are marked for exclusion) in the later stages of the review. In the case of the senior reviewer, the decrease occurs later and the recall is not as low, resulting in higher overall agreement with the baseline.

## 5 DISCUSSION

Based on the evaluation results, the potential for saving work by machine learning–powered prioritisation, as predicted by previous simulations, has been confirmed in this real‐world user study. Like the previously reported results, we have observed that the gains depend on the particular reference collection: From 7% to 71% of the screening effort could be saved. The prioritisation succeeded in suggesting relevant references across a variety of reviews from the public health domain, which has less clearly defined and more complex inclusion criteria than, for example, drug effectiveness reviews.^9^ Most importantly, the screening was conducted by the systematic reviewers from start to finish, without any intervention by the algorithm designers or modification to the software. This was possible because of the intuitive and user‐friendly Web interface for searching and organising references. However, despite this confirmation on the maturity of the tool, there are still open questions on best practices on using RobotAnalyst to realise its full potential.

The functionality provided by RobotAnalyst can be used in multiple ways beyond the start‐to‐finish screening explored in the user study. For example, the ability to prioritise and screen by clusters is an interesting scheme that may be useful for scoping reviews, or as a way to organise and assess a preliminary literature search strategy. In the case of a rapid review, 82 it may not be as essential to finish screening once enough relevant evidence has been identified. Other use‐cases include performing review updates or deciding if a review needs to be updated.^83^ For these cases, a user can upload screened references (inclusion and exclusions) from a previous review along with new references retrieved by literature search. Training and applying the classification model can prioritise the new references, and only the predicted inclusions need to be screened.

A major challenge to partial screening is the lack of a reliable threshold to stop screening. This point is ignored when computing an evaluation measure like *WSS*@95%, since the measure is based on the assumption that a user readily knows when sufficient recall has been reached; however, in reality, this is not the case. The reported *WSS*@95% serves as an upper bound on possible work savings with a stopping criterion. In practice, more screening will be necessary to achieve the same level of recall, as any stopping criterion would need to see a series consisting mainly of exclusions before signalling to stop. Deciding when to stop can be left to the end‐user, eg, if the prioritised references are consistently irrelevant, or heuristics^84^ or statistical approaches (based on an unbiased sample of the remaining references) can inform this decision. We intend to investigate this problem in the future, working in close cooperation with the systematic reviewers to ensure the solution enables them stop confidently and improve efficiency.

Another limitation of *WSS*@95% is that it measures savings in work in terms of the number of screened references, while in practice, a reviewer's time is the resource of interest. The two are not equivalent since time per decision is not constant, as we can observe in Figure 2. In prioritised (AL‐based) screening tasks, the median decision time is substantially lower in the late stages of the process. On one hand, this could be explained by the fact that the late decisions are mostly exclusions, which are easier to make. On the other hand, the prioritisation can have an impact on it as well, since having seen most of the relevant documents, a reviewer could have a better understanding of a task, which can lead to faster decisions.

Community feedback is necessary to form best practices for technology‐assisted reviews. RobotAnalyst could be incorporated without changing the screening workflow in order to alleviate some of the burden of screening large reference collections without prioritisation. The results of prioritisation can be considered useful for distributing references across a screening team.^14^ Additionally, some qualitative feedback from the evaluation indicates that users also enjoy the ability to identify relevant references earlier since they find it less mentally taxing to screen the remaining references having recognised the vast majority of the relevant references.

However, the running recall measurement shown in Figure 4 emphasise a possible danger with machine‐assisted prioritisation—depending on their experience, users may become too complacent with the machine predictions, such that references that appear later in the screening are perceived as less relevant by default, although they may meet inclusion criteria that the machine failed to recognise because of a lack of any prior examples. Thus, the user must remain vigilant throughout the screening process as, presumably, it is easier to miss a relevant reference when it is surrounded by mostly irrelevant ones.

Nonetheless, the system offers functionality for ad hoc quality assurance via random sampling to mitigate this risk. In this way, unbiased samples can be generated for checking batches of references. Alternatively, these random samples could be used to gauge the level of specificity in the collection and estimate recall. The functionality could also be extended to track the time spent screening each reference, which is essential to assess cost‐effectiveness of systematic reviews.^7^

Our evaluation has been conducted on primarily public health review questions. Public health questions are complex, involving behaviour, culture, and organisations, and often need to be described using abstract, fuzzy terminology. Screening in this setting is arguably more challenging than with clinical research questions, which may have more well‐defined populations, interventions, comparators, and outcomes. Techniques like query expansion, topic modelling, and descriptive clustering can help explore terminological variation, and machine learning can handle diverse vocabularies.

Our evaluation of descriptive clustering and topic modelling reported in Appendix B focuses on the coherence of these organisation techniques and whether the descriptions were meaningful. Our hypothesis is that a user could use the clustering to find relevant references when beginning screening—an assumption that has been confirmed for topic modelling but needs evaluation for descriptive clustering. Alternatively, RobotAnalyst's search capabilities allow a user to perform a focused search via keywords, clusters, or topics, to find relevant references that may have distinct vocabulary and are not being prioritised by the model. That is, focused and topical search capabilities may be crucial for initialisation and to ensure a complete coverage when used in conjunction with the automatic prioritisation. Future works should consider a controlled study of the impact of the initial choice of references on the active learning performance. This would require the same review to be initiated several times by independent users, each using randomly assigned search strategies (keyword, cluster, or topic based) within a collection.

While this work used a single model to prioritise references, it may be useful to explore the case of multiple models for different PICO elements or inclusion criteria. Even in public health, user feedback from NICE reviewers indicated that while the system was found to perform very well for single PICO (ie, singular review question) screening, there was room for improvement with collections covering multiple review questions.

Evaluating the performance of RobotAnalyst for focused clinical reviews is another direction of future work. For reviews with clear PICO‐based criteria, it may be necessary to use features with more clinical specificity such as MeSH terms or automatically recognised entities such as drug names, proteins, and genes. Using information extraction techniques for targeting such entities in full article text would support inclusion criteria that cannot be verified based only on the abstract. Using full text provides more content for the classification model, clustering, and topic modelling. However, computational processing and storage would be significantly higher for full text. Furthermore, processing full text is challenging because of access costs, copyright limitations on third‐party processing and storage, and the technical challenges of reliably extracting text from PDF documents. To achieve uniform screening, these issues would have to be overcome for every reference in a collection.

In summary, this study confirms that references can be reliably prioritised in public health and points to the potential benefit of incorporating tools such as search within collection, topic modelling, and descriptive clustering to aid initial screening or to ensure coverage of a collection. The study has a number of limitations including the following:

- The potential work savings is an upper bound on what is achievable in reality. Reducing the number of screening references requires a stopping criterion and the associated risk of missed relevancy, which may be unacceptable in certain cases.
- In terms of the time spent screening, the savings may be higher or lower than what would be indicated by the work saved in terms of the number of references, since the screening times vary per decision and throughout the process.
- Issues with complacency may be inflating the work savings estimates, if late in the screening process truly relevant references were missed.
- Further study is needed to compare using descriptive clustering, topic modelling, and keyword search to find the initial set of references before active learning prioritisation.
- The evaluation primarily centred on public health reviews. It would be worthwhile to evaluate the work savings in other review domains.

Further research and evaluation studies can address several of these points. To be efficient, the studies should be performed on prospective reviews that need multiple screenings to facilitate paired comparisons.

Machine‐assisted prioritisation for systematic reviews is a paradigm shift away from the traditional manual labour intensive approach. The potential time savings of using prioritisation are considerable for large collections with low specificity. The systematic review community needs to embrace the new technology to improve efficiency,^4^ and support further innovation through participation and community feedback.

## 6 CONCLUSION

We have presented a description and evaluation of RobotAnalyst as a tool to screen and organise references for systematic reviews on public health and health services research topics. The evaluation was the first of its kind in terms of multiple new reviews completed from start to finish within RobotAnalyst. The results indicate that substantial gains can be made by using machine learning to actively prioritise relevant references. The promising results for descriptive clustering highlight another avenue for exploring large reference collections. Currently, RobotAnalyst provides functionality for searching within a collection and browsing subsets of the results using semantic similarity based on terminology or topics. These new interfaces may help reviewers screen large collections of disparate references arising from complex review questions. While it is possible to extend and enhance the functionality, there is ample evidence to suggest that machine learning techniques for prioritising references in systematic reviews have matured with multiple systems available to end‐users. More prospective evaluations and open discussions are needed to spur the community to adopt tools like RobotAnalyst as the default, rather than the exception.

## Figures and Tables

**Figure A1:**
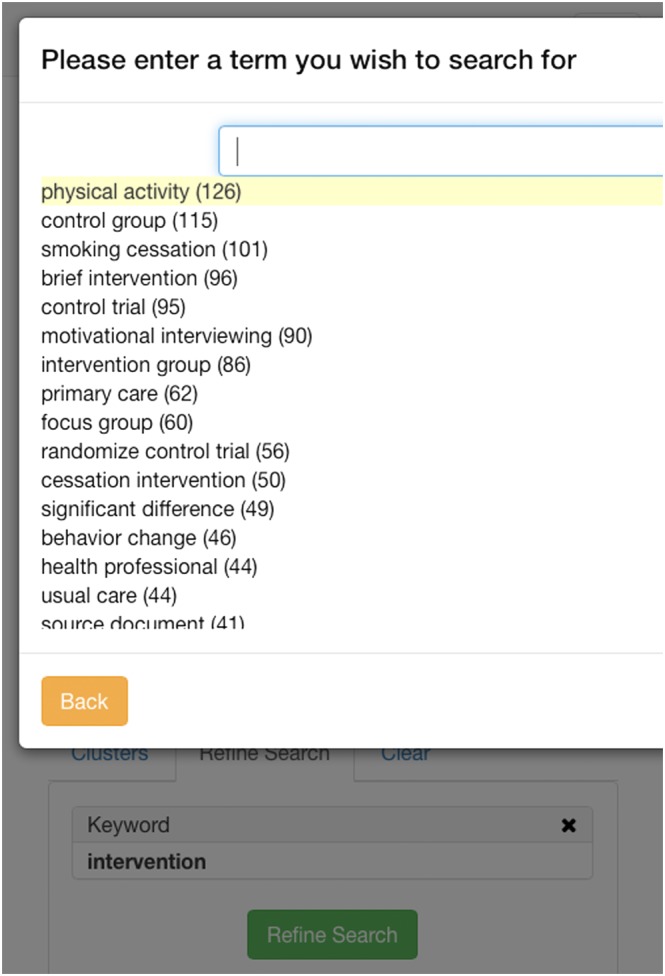
A screenshot of the search interface with faceting. A user has searched for references that contain the word intervention and is exploring further possible refinement based on extracted terms

**Figure A2:**
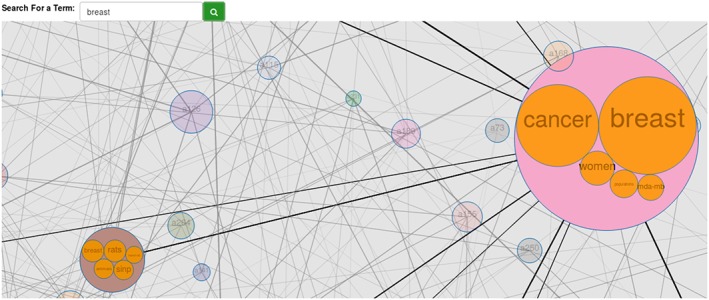
Topic visualisation in RobotAnalyst. A part of the topic visualisation generated for a collection of references related to cancer. A query for the word breast has highlighted two groups: one corresponding to human breast cancer (described by breast, cancer, and women) and one corresponding to animal studies (described by rats, sinp, breast, and animals). A link between the two circles means that the topics are related

**Figure A3:**
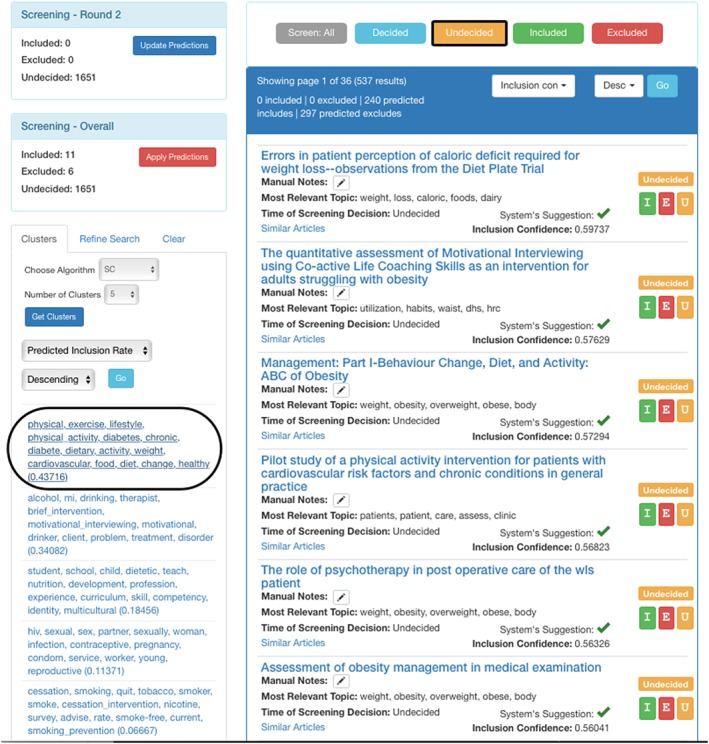
A screenshot of the interface with prioritised clusters. A user has elected to screen only the undecided references within the first cluster (encircled) with the results prioritised by inclusion confidence. The descriptive label for this cluster is “physical, exercise, lifestyle, physical activity, diabetes, chronic, diabete, dietary, activity, weight, cardiovascular, food, diet, change, healthy,” where the underlined term is automatically extracted by Termine.

**Figure B1:**
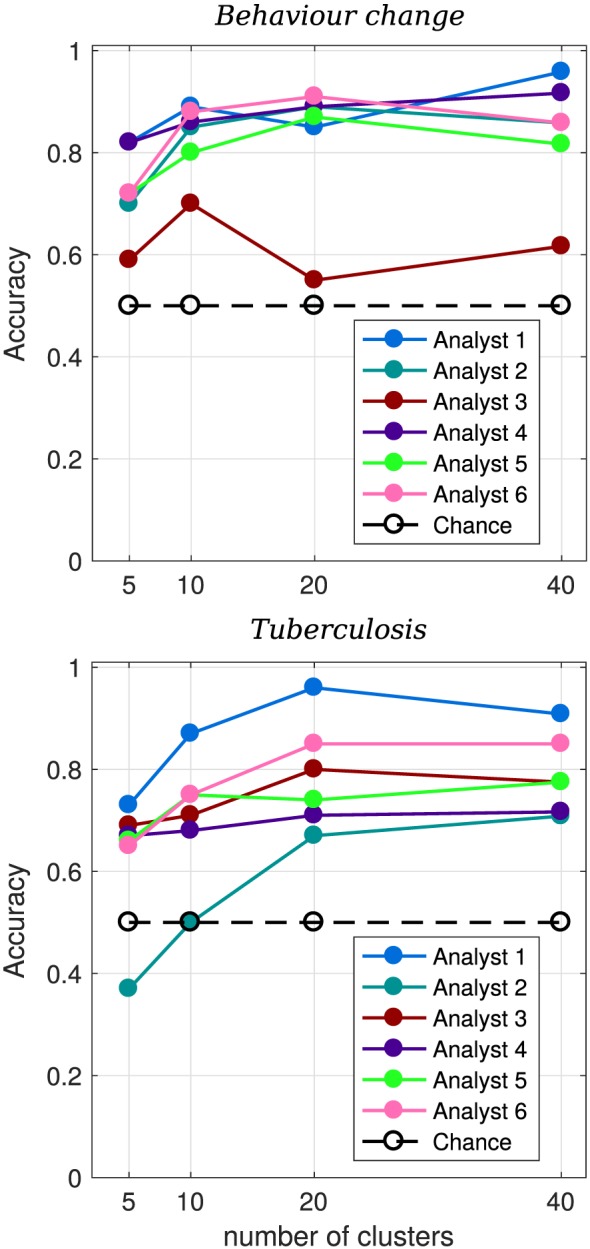
Descriptive clustering outlier detection accuracy for different numbers of clusters in two reference collections. Tasks consisted of determining which of two references matches the cluster description

**Figure B2:**
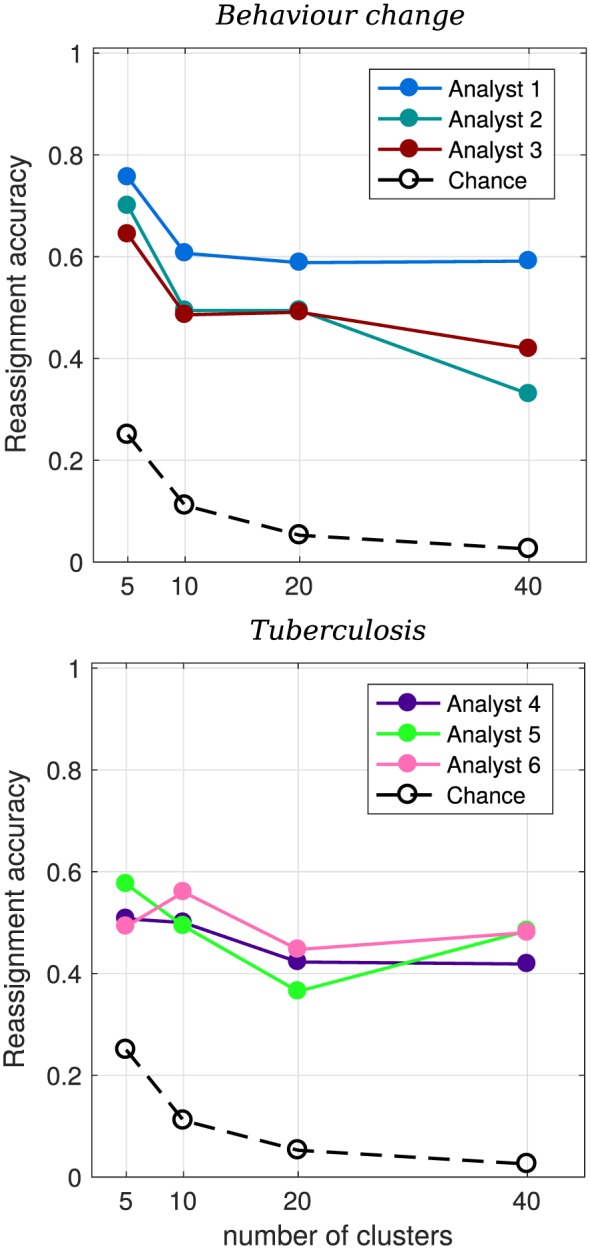
Descriptive clustering outlier reassignment accuracy across an increasing number of clusters in two reference collections. The accuracy is the proportion of recognised outliers which were assigned to their original cluster correctly based on the cluster descriptions

## APPENDIX A SYSTEM INTERFACE

### A.1

In this appendix, we describe RobotAnalyst's interface for searching, screening, and exporting reference collections.

#### A.1 Faceted search

To support reviewers seeking specific information, RobotAnalyst provides a search interface for selecting and analysing a portion of references and for understanding the overall thematic content. The search interface allows a reviewer to choose references based on metadata, such as author or journal name, and content (title and abstract). Content can be searched using individual words or multiword terms retrieved by Termine. The search interface supports faceting, which means that a reviewer can search within results of a previous search. The interface supports this iterative process by displaying the most relevant search terms within the current query and calculating how many references the refinement would yield. An example search query is shown in Figure A1.

#### A.2 Topic model interface

RobotAnalyst's topic‐based search tool presents a visualisation of the entire collection as a mixture of topics as shown in Figure A2. Each topic is represented by a circle whose size is proportional to its prevalence in the collection. The topics are described by the five words with the strongest association with the topic; this enables a user to search for topics containing a relevant word. Alternatively, a user can explore similar topics by following the lines that connect topic circles.

Once a topic is chosen, RobotAnalyst selects the references most focused on the topic. RobotAnalyst provides a fine‐grained model with 300 topics.

#### A.3 Clustering interface

The descriptive clustering interface provides an automatic organisation of a reference collection into a smaller number of clusters (from 5 to 100 clusters). Each cluster is described by a list of typical words and terms. Reviewers can select the number of clusters to be a small number to see a rough categorisation of the different themes within the collection, or select more clusters to divide the collection into numerous fine‐grained, but coherent, groups of references. Once a classification model has been trained the clusters can be sorted by the proportion of predicted inclusions. This enables a cluster‐based screening approach that allows reviewers to concentrate on a relevant cluster of references. An example is shown in Figure A3. The faceted search can be used in conjunction with cluster‐based search to form precise queries.

#### A.4 Screening interface

With or without the functionality described above, a user can screen the references displayed in the results pane. Each reference is shown with its current relevancy status. Once a classification model has been trained, the system's prediction and inclusion confidence are shown for each reference. Based on these data and inclusion criteria of the review, the user can make the decision on whether to include or exclude the reference or mark it as undecided. The interface is designed to facilitate the rapid processing of references, while recording automatically information about the decision time and prioritisation method.

For collections with screening decisions, the search queries can be combined with filters for the status of the screening decisions. For example, a reviewer can restrict the search to those that have already been manually included, or find the references in a cluster that remain to be screened.

The search functionality can be used by a user to ensure coverage of the relevancy that may be missed by the model. For example, if the screened instances all belong to an isolated cluster, then they will not assist in recognising and prioritising relevant references from another cluster with distinct vocabulary. After exhausting the relevant instances within the initial cluster, the user could re‐examine the set of clusters looking for another cluster that could be relevant. This process could continue across multiple clusters. With this approach, clustering can be used to explore the space to ensure coverage of all themes.

#### A.5 Export

RobotAnalyst allows exporting subsets of references in the RIS file format to be used with other software. Any screening decisions and manually entered notes are saved on export as additional fields that will be recognised on import back into RobotAnalyst. This enables reviewers to share screening decisions, or to use previous screening decisions to facilitate a review update.

## APPENDIX B DESCRIPTIVE CLUSTERING EVALUATION

### B.1

To ensure that the descriptive clustering organises references into meaningful clusters and provides informative keyword lists, we conducted an evaluation that was performed by reviewers from NICE. Evaluation tasks were created for two reference collections from NICE guidelines, *“Behaviour change: individual approaches”* and “Tuberculosis,” the same collections used in the screening evaluation task.

The first set of tasks assessed the coherence of clusters by testing whether a reviewer could distinguish an outlier reference inserted into a set of another cluster's prototypical references. The second set of tasks assessed whether a user could distinguish a random outlier reference from a random reference from within a cluster given its description, and then predict the outlier's true correct cluster membership. The task designs are based on the evaluation of Lau et al for topic models^85^ but are adapted for clustering rather than latent mixture models.

For the first set of tasks, the provided instructions stated, “Each task consists of identifying which reference, out of a set of four, does not fit the theme of the cluster. The theme of the cluster should be apparent from the cluster description and the other three references.” The reviewers were also instructed to skip tasks without a clear outlier rather than guess. The tasks were generated for both collections with 20 clusters and five tasks per cluster. For each cluster, the 25 most prototypical references were kept, and the outliers were assigned by randomly permuting five of these references to other clusters. A cluster's prototypical references were chosen as those nearest to the cluster's centroid. Essentially, this task assessed whether a prototypical reference from one cluster could be distinguished from a group of prototypical references of another cluster given its keywords. The chance rate for random guessing on this task is 20%.

The descriptive clustering algorithm described in Section 3.2 was used to form the clusters and select the keywords. For this task, the number of keywords was selected such that the keyword list was not more than 150 characters long (including commas and spaces). For comparison, cluster assignments were obtained from the LDA topic model described in Section 3.2 by choosing the 20 most prevalent topics, then assigning each reference to a cluster corresponding to the topic with the highest relevance, and using this same relevance to rank the references within the cluster.

Three reviewers performed the tasks for “Behaviour change” and three other reviewers performed the tasks for “Tuberculosis.” The reviewers were blinded to the name of the method (descriptive clustering or topic modelling). The outlier detection accuracy for each reviewer is presented in Table A1. The accuracy was significantly higher (significance level of 0.05) for spectral clustering versus topic modelling across the *n*  =  6 users (one‐sided sign‐rank test with *P*value of 0.015625). Per user, outlier detection accuracy is always higher for spectral clustering versus topic modelling.

**Table A1:** Descriptive clustering outlier detection accuracy of six reviewers split between two collections (the average accuracy per collection in parentheses)^a^

	*Behaviour change*				*Tuberculosis*			
Spectral clustering	75%	69%	92%	(78.67%)	49%	83%	69%	(67%)
Topic modelling	63%	15%	75%	(51%)	30%	58%	38%	(42%)

^*a*^Reviewers used any apparent coherence of the references and the description to choose an outlier reference. Accuracy is computed for 100 tasks with a chance rate of 20%.

The difference in accuracy demonstrates that spectral clustering provided a more coherent organisation of a collection versus topic modelling, since reviewers were able to distinguish outlier references more easily.

Based on the previous results, a second set of tasks involving only spectral clustering was performed in order to check whether the cluster descriptions were sufficiently accurate to enable a user to predict the cluster membership of specific references as the number of clusters was varied across 5, 10, 20, and 40 clusters. Tasks were performed in two stages: stage A, which was outlier detection (as before but without restriction to prototypical references), and stage B, which was cluster reassignment (selecting the outlier's original cluster). There were 100 tasks for the case of 5, 10, and 20 clusters with 120 tasks for the 40 cluster case; tasks were divided evenly between the clusters.

For stage A, the outlier detection tasks consisted of determining which of two references did not match the provided keyword list. With only two choices, this task assessed only the descriptiveness of the keywords and not the cluster coherence. The within‐cluster reference was drawn uniformly without replacement, as was the outlier. The expected random performance for this task is 50% accuracy. Six reviewers performed the tasks for both collections. The results for each reviewer across the number of clusters are shown in Figure B1. The accuracy increases in most cases and does not decrease markedly with more clusters, which indicates that the descriptive clustering can be used effectively across this range.

In stage B, the reviewers performed cluster reassignment for the outlier reference: They revisited each outlier task and had to decide which of the other clusters best matches the excluded reference. To facilitate this match, the full list of cluster descriptions was provided as a separate document outside the RobotAnalyst interface. Reviewers entered their decision using RobotAnalyst's manual note field. Three reviewers completed this task for each collection. Decisions that matched the outlier's original cluster are counted as correct. Cases where a reviewer entered multiple clusters for one reference were counted as incorrect. Performance is assessed in terms of the reassignment accuracy for tasks completed correctly in stage A. The expected random performance for this task is 1/(k − 1), where k is the number of clusters. The results are shown in Figure B2.

Although the reassignment accuracy decreases when there are more than 5 clusters, it is above the 99% confidence bound for random guessing in all cases. The reference‐cluster assignment accuracy is above 35% for 40 clusters (chance rate of 2.56%) for all of the users and collections. This indicates that, in over one third of the cases, users are able to use the descriptions to predict precisely to which cluster a reference belongs, but there remains a majority of possibly ambiguous references that are difficult to localise to a particular cluster.

## References

[1] Higgins JP , Deeks JJ . Selecting studies and collecting data; 2011. Version 5.1.0 [updated March 2011].

[2] NICE | The National Institute for Health and Care Excellence. https://www.nice.org.uk; Accessed November 11, 2017.

[3] The Campbell Collaboration. http://www.campbellcollaboration.org. Accessed November 11, 2017.

[4] Bastian H , Glasziou P , Chalmers I . Seventy‐five trials and eleven systematic reviews a day: how will we ever keep up? PLoS Med. 2010;7(9):e1000326. 20877712 10.1371/journal.pmed.1000326 PMC2943439

[5] Edwards P , Clarke M , DiGuiseppi C , Pratap S , Roberts I , Wentz R . Identification of randomized controlled trials in systematic reviews: accuracy and reliability of screening records. Stat Med. 2002;21(11):1635‐1640. 12111924 10.1002/sim.1190

[6] Bramer WM , Milic J , Mast F . Reviewing retrieved references for inclusion in systematic reviews using EndNote. J Med Libr Assoc. 2017;105(1):84‐87. 28096751 10.5195/jmla.2017.111 PMC5234463

[7] Shemilt I , Khan N , Park S , Thomas J . Use of cost‐effectiveness analysis to compare the efficiency of study identification methods in systematic reviews. Syst Rev. 2016;5(140). 10.1186/s13643-016-0315-4 PMC4989498 27535658

[8] Lefebvre C , Glanville J , Wieland LS , Coles B , Weightman AL . Methodological developments in searching for studies for systematic reviews: past, present and future? Syst Rev. 2013;2(78). 10.1186/2046-4053-2-78 PMC4015986 24066664

[9] Cohen AM , Hersh WR , Peterson K , Yen PY . Reducing workload in systematic review preparation using automated citation classification. J Am Med Inform Assoc. 2006;13(2):206‐219. 16357352 10.1197/jamia.M1929 PMC1447545

[10] Elliott JH , Turner T , Clavisi O , et al. Living systematic reviews: an emerging opportunity to narrow the evidence‐practice gap. PLoS Med. 2014;11(2):e1001603. 24558353 10.1371/journal.pmed.1001603 PMC3928029

[11] Thomas J , Noel‐Storr A , Marshall I , et al. Living systematic reviews: 2. Combining human and machine effort. J Clin Epidemiol. 2017;91. 10.1016/j.jclinepi.2017.08.011 28912003

[12] Cohen AM , Ambert K , McDonagh M . Studying the potential impact of automated document classification on scheduling a systematic review update. BMC Med Inform Decis Mak. 2012;12(1):33. 22515596 10.1186/1472-6947-12-33 PMC3420236

[13] Wallace BC , Small K , Brodley CE , et al. Toward modernizing the systematic review pipeline in genetics: efficient updating via data mining. Genet Med. 2012;14(7):663‐669. 22481134 10.1038/gim.2012.7 PMC3908550

[14] O'Mara‐Eves A , Thomas J , McNaught J , Miwa M , Ananiadou S . Using text mining for study identification in systematic reviews: a systematic review of current approaches. Syst Rev. 2015;4(5). 10.1186/2046-4053-4-5 PMC4320539 25588314

[15] Cohen AM . Optimizing feature representation for automated systematic review work prioritization. In: AMIA Annual Symposium Proceedings, Vol. 2008; 2008:121‐125. PMC2656096 18998798

[16] Bekhuis T , Demner‐Fushman D . Towards automating the initial screening phase of a systematic review. In: World congress on medical informatics (medinfo) Safran C, Reti S, Marin HF, eds., Stud Health Technol Inform, vol. 160; 2010:146‐150. 20841667

[17] Bekhuis T , Demner‐Fushman D . Screening nonrandomized studies for medical systematic reviews: a comparative study of classifiers. Artif Intell Med. 2012;55(3):197‐207. 22677493 10.1016/j.artmed.2012.05.002 PMC3393813

[18] Bekhuis T , Tseytlin E , Mitchell KJ , Demner‐Fushman D . Feature engineering and a proposed decision‐support system for systematic reviewers of medical evidence. PLOS ONE. 2014;9(1):e86277. 24475099 10.1371/journal.pone.0086277 PMC3903545

[19] Matwin S , Kouznetsov A , Inkpen D , Frunza O , O'Blenis P . A new algorithm for reducing the workload of experts in performing systematic reviews. J Am Med Inform Assoc. 2010;17(4):446‐53. 20595313 10.1136/jamia.2010.004325 PMC2995653

[20] Frunza O , Inkpen D , Matwin S . Building systematic reviews using automatic text classification techniques. In: International Conference on Computational Linguistics; 2010:303‐311.

[21] Frunza O , Inkpen D , Matwin S , Klement W , O'Blenis P . Exploiting the systematic review protocol for classification of medical abstracts. Artif Intell Med. 2011;51(1):17‐25. 21084178 10.1016/j.artmed.2010.10.005

[22] Wallace BC , Trikalinos TA , Lau J , Brodley C , Schmid CH . Semi‐automated screening of biomedical citations for systematic reviews. BMC Bioinf. 2010;11(1):55. 10.1186/1471-2105-11-55 PMC2824679 20102628

[23] Small K , Wallace B , Trikalinos T , Brodley CE . The constrained weight space SVM: learning with ranked features. In: International conference on machine learning (ICML); 2011:865‐872.

[24] Jonnalagadda S , Petitti D . A new iterative method to reduce workload in systematic review process. Int J Comput Biol Drug Des. 2013;6(1‐2):5‐17. 23428470 10.1504/IJCBDD.2013.052198 PMC3787693

[25] Dalal SR , Shekelle PG , Hempel S , Newberry SJ , Motala A , Shetty KD . A pilot study using machine learning and domain knowledge to facilitate comparative effectiveness review updating. Med Decis Making. 2013;33(3):343‐355. 22961102 10.1177/0272989X12457243

[26] Miwa M , Thomas J , O'Mara‐Eves A , Ananiadou S . Reducing systematic review workload through certainty‐based screening. J Biomed Inform. 2014;51:242‐53. 24954015 10.1016/j.jbi.2014.06.005 PMC4199186

[27] Timsina P , Liu J , El‐Gayar O . Advanced analytics for the automation of medical systematic reviews. Inf Syst Front. 2016;18(2):237‐252.

[28] Khabsa M , Elmagarmid A , Ilyas I , Hammady H , Ouzzani M . Learning to identify relevant studies for systematic reviews using random forest and external information. Mach Learn. 2016;102(3):465‐482.

[29] Hashimoto K , Kontonatsios G , Miwa M , Ananiadou S . Topic detection using paragraph vectors to support active learning in systematic reviews. J Biomed Inform. 2016;62:59‐65. 27293211 10.1016/j.jbi.2016.06.001 PMC4981645

[30] Liu J , Timsina P , El‐Gayar O . A comparative analysis of semi‐supervised learning: the case of article selection for medical systematic reviews. Inf Syst Front. 2016:1–.

[31] Kontonatsios G , Brockmeier AJ , Przybyła P , McNaught J , Mu T , Goulermas JY , Ananiadou S . A semi‐supervised approach using label propagation to support citation screening. J Biomed Inform. 2017;72:67‐76. 28648605 10.1016/j.jbi.2017.06.018 PMC5726085

[32] Cohen AM , Ambert K , McDonagh M . A prospective evaluation of an automated classification system to support evidence‐based medicine and systematic review. In: AMIA Annual Symposium Proceedings, Vol. 2010; 2010:121. PMC3041348 21346953

[33] Wallace BC , Small K , Brodley CE , Lau J , Trikalinos Ta . Deploying an interactive machine learning system in an evidence‐based practice center: abstrackr In: ACM SIGHIT Symposium on International Health Informatics. 2012:819.

[34] Thomas J , Brunton J , Graziosi S . EPPI‐Reviewer 4.0: Software for Research Synthesis. London: EPPI‐Centre, Social Science Research Unit, Institute of Education, University of London; 2011.

[35] Ouzzani M , Hammady H , Fedorowicz Z , Elmagarmid A . Rayyan‐a web and mobile app for systematic reviews. Syst Rev. 2016;5(210). 10.1186/s13643-016-0384-4 PMC5139140 27919275

[36] Howard BE , Phillips J , Miller K , et al. SWIFT‐review: a text‐mining workbench for systematic review. Syst Rev. 2016;5(87). 10.1186/s13643-016-0263-z PMC4877757 27216467

[37] Khodambashi S , Nytrø Ø . A systematic literature review on evaluation of digital tools for authoring evidence‐based clinical guidelines. In: Australian National Health Informatics Conference (HIC), 2017;239:48. 28756436

[38] Cutting DR , Karger DR , Pedersen JO , Tukey JW . Scatter/gather: a cluster‐based approach to browsing large document collections. In: ACM SIGIR Conference on Research and Development in Information Retrieval; 1992:318‐329.

[39] Carpineto C , Osiński S , Romano G , Weiss D . A survey of web clustering engines. ACM Comput Surv. 2009;41(3):1‐38.

[40] Mu T , Goulermas JY , Korkontzelos I , Ananiadou S . Descriptive document clustering via discriminant learning in a co‐embedded space of multilevel similarities. J Assoc Inf Sci Technol. 2016;67(1):106‐33.

[41] Brockmeier AJ , Mu T , Ananiadou S , Goulermas JY . Self‐tuned descriptive document clustering using a predictive network. IEEE Trans Knowl Data Eng. https://doi.org./10.1109/TKDE.2017.2781721

[42] Hofmann T . Probabilistic latent semantic indexing. In: ACM SIGIR conference on research and development in information retrieval; 1999:50‐57.

[43] Blei DM , Ng AY , Jordan MI . Latent Dirichlet allocation. J Mach Learn Res. 2003;3:993‐1022.

[44] Griffiths TL , Steyvers M . Finding scientific topics. Proc Natl Acad Sci. 2004;101(suppl 1):5228‐5235. 14872004 10.1073/pnas.0307752101 PMC387300

[45] Soleimani H , Miller DJ . Parsimonious topic models with salient word discovery. IEEE Trans Knowl Data Eng. 2015;27(3):824‐837.

[46] Mei Q , Shen X , Zhai C . Automatic labeling of multinomial topic models; 2007:490‐99.

[47] Wilbur WJ . A thematic analysis of the AIDS literature. In: Pacific Symposium on Biocomputing; 2001:386‐397. 11928492

[48] Hearst MA , Pedersen JO . Reexamining the cluster hypothesis: scatter/gather on retrieval results. In: ACM SIGIR Conference on Research and Development in Information Retrieval; 1996:76‐84.

[49] Aletras N , Baldwin T , Lau JH , Stevenson M . Representing topics labels for exploring digital libraries. In: ACM/IEEE-CS Joint Conference on Digital Libraries; 2014:239‐248.

[50] Aphinyanaphongs Y , Aliferis CF . Text categorization models for retrieval of high quality articles in internal medicine. In: AMIA annual symposium proceedings, Vol. 2003; 2003:31‐35. PMC1480096 14728128

[51] Aphinyanaphongs Y , Tsamardinos I , Statnikov A , Hardin D , Aliferis CF . Text categorization models for high‐quality article retrieval in internal medicine. J Am Med Inform Assoc. 2005;12(2):207‐216. 15561789 10.1197/jamia.M1641 PMC551552

[52] Haynes RB , Wilczynski N , McKibbon KA , Walker CJ , Sinclair JC . Developing optimal search strategies for detecting clinically sound studies in medline. J Am Med Inform Assoc. 1994;1(6):447‐458. 7850570 10.1136/jamia.1994.95153434 PMC116228

[53] Cohen AM , Ambert K , McDonagh M . Cross‐topic learning for work prioritization in systematic review creation and update. J Am Med Inform Assoc. 2009;16(5):690‐704. 19567792 10.1197/jamia.M3162 PMC2744720

[54] Lewis DD , Gale WA . A sequential algorithm for training text classifiers. In: ACM SIGIR conference on research and development in information retrieval; 1994:3‐12.

[55] Tong S , Koller D . Support vector machine active learning with applications to text classification. J Mach Learn Res. 2001;2:45‐66.

[56] Settles B . Active learning literature survey. *Computer Sciences Technical Report*, University of Wisconsin–Madison; 2009.

[57] Salton G , Buckley C . Improving retrieval performance by relevance feedback. J Assoc Inf Sci Technol. 1990;41(4):288‐297.

[58] Frantzi K , Ananiadou S , Mima H . Automatic recognition of multi‐word terms: the C‐value/NC‐value method. Int J Digital Libr. 2000;3(2):115‐130.

[59] Shemilt I , Simon A , Hollands GJ , et al. Pinpointing needles in giant haystacks: use of text mining to reduce impractical screening workload in extremely large scoping reviews. Res Syn Meth. 2014;5(1):31‐49. 10.1002/jrsm.1093 26054024

[60] Donmez P , Carbonell JG . Proactive learning: cost‐sensitive active learning with multiple imperfect oracles; 2008:619‐628.

[61] Nguyen AT , Wallace BC , Lease M . Combining crowd and expert labels using decision theoretic active learning. In: AAAI Conference on Human Computation and Crowdsourcing; 2015.

[62] Rathbone J , Hoffmann T , Glasziou P . Faster title and abstract screening? Evaluating Abstrackr, a semi‐automated online screening program for systematic reviewers. Syst Rev. 2015;4(80). 10.1186/s13643-015-0067-6 PMC4472176 26073974

[63] McCallum AK . MALLET: A machine learning for language toolkit. 2002.

[64] Mikolov T , Sutskever I , Chen K , Corrado GS , Dean J . Distributed representations of words and phrases and their compositionality; In: Advances in Neural Information Processing Systems; 2013:3111‐119.

[65] Pennington Jeffrey , Socher Richard , Manning ChristopherD . Glove: global vectors for word representation. In: Empirical Methods in Natural Language Processing (EMNLP); 2014:1532‐1543.

[66] Ananiadou S , Rea B , Okazaki N , Procter R , Thomas J . Supporting systematic reviews using text mining. Soc Sci Comput Rev. 2009;27(4):509‐523.

[67] Choi S , Ryu B , Yoo S , Choi J . Combining relevancy and methodological quality into a single ranking for evidence‐based medicine. Inf Sci. 2012;214:76‐90.

[68] Marshall IJ , Kuiper J , Wallace BC . RobotReviewer: evaluation of a system for automatically assessing bias in clinical trials. J Am Med Inform Assoc. 2015;23(1):193‐01. 26104742 10.1093/jamia/ocv044 PMC4713900

[69] Cohen AM , Smalheiser NR , McDonagh MS , Yu C , Adams CE , Davis JM , Yu PS . Automated confidence ranked classification of randomized controlled trial articles: an aid to evidence‐based medicine. J Am Med Inform Assoc. 2015;22(3):707‐717. 25656516 10.1093/jamia/ocu025 PMC4457112

[70] Millard LA , Flach PA , Higgins JP . Machine learning to assist risk‐of‐bias assessments in systematic reviews. Int J Epidemiol. 2015;45(1):266‐77. 26659355 10.1093/ije/dyv306 PMC4795562

[71] Marshall IJ , Noel‐Storr A , Kuiper J , Thomas J , Wallace BC. Machine learning for identifying randomized controlled trials: an evaluation and practitioner's guide. Res Syn Meth. 2018. 10.1002/jrsm.1287 PMC6030513 29314757

[72] Wallace BC , Marshall IJ . Extracting PICO sentences from clinical trial reports using supervised distant supervision. J Mach Learn Res. 2016;17:1‐25. PMC5065023 27746703

[73] “RIS” format documentation. Thomson Reuters ‐ ResearchSoft http://endnote.com/sites/rm/files/m/direct_export_ris.pdf; 2008.

[74] Tsuruoka Y , Tateishi Y , Kim JD , et al. Developing a robust part‐of‐speech tagger for biomedical text, Vol. 3746 LNCS; 2005:382‐392.

[75] Ng AY , Jordan MI , Weiss Y . On spectral clustering: analysis and an algorithm. In: Advances in Neural Information Processing Systems, Vol. 14; 2002:849‐56.

[76] Salton G , Wong A , Yang CS . A vector space model for automatic indexing. Commun ACM. 1975;18(11):613‐620.

[77] Bahmani B , Moseley B , Vattani A , Kumar R , Vassilvitskii S . Scalable k‐means++. VLDB Endowment. 2012;5(7):622‐633.

[78] Fleuret F . Fast binary feature selection with conditional mutual information. J Mach Learn Res. 2004;5(11):1531‐1555.

[79] Schwarz G . Estimating the dimension of a model. Ann Stat. 1978;6(2):461‐464.

[80] Fan RE , Chang KW , Hsieh CJ , Wang XR , Lin CJ . LIBLINEAR: a library for large linear classification. J Mach Learn Res. 2008;9:1871‐1874.

[81] Kanoulas E , Li D , Azzopardi L , Spijker R . CLEF 2017 technologically assisted reviews in empirical medicine overview. In: Working notes of CLEF 2017 ‐ conference and labs of the evaluation forum Cappellato L, Ferro N, Goeuriot L, Mandl T, eds., CEUR Workshop Proceedings, vol. 1866; 2017.

[82] Wagner G , Nussbaumer‐Streit B , Greimel J , Ciapponi A , Gartlehner G . Trading certainty for speed—how much uncertainty are decisionmakers and guideline developers willing to accept when using rapid reviews: an international survey. BMC Med Res Methodol. 2017;17(1):121. 28806999 10.1186/s12874-017-0406-5 PMC5557322

[83] Garner Paul , Hopewell Sally , Chandler Jackie , et al. When and how to update systematic reviews: consensus and checklist. BMJ. 2016;354:i3507. 27443385 10.1136/bmj.i3507 PMC4955793

[84] Cormack GV , Grossman MR . Engineering quality and reliability in technology‐assisted review. In: ACM SIGIR Conference on Research and Development in Information Retrieval; 2016:75‐84.

[85] Lau JH , Newman D , Baldwin T . Machine reading tea leaves: automatically evaluating topic coherence and topic model quality. In: Conference of the European Chapter of the Association for Computational Linguistics (EACL); 2014April:530‐539.

